# Vacuolating Cytotoxin A (VacA) and Extracellular Vesicles in *Helicobacter pylori*: Two Key Arms in Disease Development 

**DOI:** 10.30699/ijp.2024.2031417.3312

**Published:** 2024-01-10

**Authors:** Seyedeh Faride Alavi Rostami, Mansoor Khaledi, Fatemeh Dalilian, Mahtab Mehboodi, Atefeh Akbari, Milad Shahini Shams Abadi, Pouria Khodaei Ataloo, Zeinab Mohsenipour, Samad Rastmanesh

**Affiliations:** 1 *Department of Microbiology, Faculty of Biological Sciences, Islamic Azad University Tehran-North Branch, Tehran, Iran*; 2 *Department of Microbiology and Immunology, School of Medicine, Shahrekord University of Medical Sciences, Shahrekord, Iran*; 3 *Cellular and Molecular Research Center, Basic Health Sciences Institute, Shahrekord University of Medical Sciences, Shahrekord, Iran*; 4 *Department of Microbiology, Mashhad University of Bacic science, Mashhad, Iran*; 5 *Department of Medical Microbiology (Bacteriology & Virology), Afzalipour Faculty of Medicine, Kerman University of Medical Sciences, Kerman, Iran*; 6 *Department of Microbiology, Faculty of Science, Qom branch, Islamic Azad University, Qom, Iran*; 7 *Department of Microbiology, School of Medicine, Ardabil University of Medical Science, Ardabil, Iran*; 8 *Department of Microbiology, School of Medicine, Tehran University of Medical Sciences, Tehran, Iran*; 9 *Department of Bacteriology and Virology, School of Medicine, Tabriz University of Medical Science, Tabriz, Iran*; # * Authors considered as first author*

**Keywords:** Autophagy, Cell signaling, Disease development, Extracellular vesicles, Immune cells, Helicobacter disease

## Abstract

Extracellular vesicles (EVs) are cell-derived vesicles that play a critical role in host-pathogen interactions, facilitating intercellular communication and transporting both pathogen- and host-derived molecules during infection spread. To regulate their environment, for instance, by modulating innate and adaptive inflammatory immune responses, pathogens may alter the composition of EVs produced by infected cells. Gastric cancer is one of the leading causes of cancer-related deaths worldwide, and Helicobacter pylori infection is considered a significant risk factor for its development. This cancer is characterized by significant inflammation mediated by EVs generated from infected host cells. H. pylori contributes substantially to inflammation, promoting disease progression. Moreover, H. pylori produces and releases vesicles known as outer membrane vesicles (H. pylori-OMVs), which contribute to the shrinkage and cellular transformation of the gastric epithelium. Although the vacuolating cytotoxin A (VacA) plays a critical role in pathogenesis, its association with EVs in H. pylori has not been previously addressed. Understanding the roles of extracellular vesicles and VacA during H. pylori infection—whether they benefit the host or the pathogen—could pave the way for new treatment approaches. This review briefly discusses the role of VacA and extracellular vesicles in the growth and pathogenesis of *H. pylori*.

## Introduction

Spiral-shaped gram-negative microaerophile *Helicobacter pylori (H. pylori)* is a member of the *Helicobacteriacea* family and the *Campylobacterales* order of the *Proteobacteria*. They colonize the human gastric mucosa and cause gastritis, peptic ulcer, cancer and even extra-gastric disorders (1-3). The considerable genetic diversity of these bacteria is what contributes to their pathogenicity and capacity to cause a number of diseases (4).

Geographical location or habitational circumstances influence the prevalence of *H. pylori* infection. Additionally, as human populations fluctuate, new genetic variations of *H. pylori* appear (5-7). Escape from the immune system occurs due to motility, diversity of adhesion, urease production, expression of Lewis (Le) blood group antigens (Lex and Ley) and various effective molecules in *H. pylori*. Therefore, this bacterium adapts well to the challenging environment of the stomach (2, 3, 8). *H. Pylori* reduces host immune responses during the chronic phase of infection while *H. pylori* overcome the oxidative damage brought on by the overwhelming inflammatory response during the acute phase of colonization. And promote tolerance rather than a protective reaction in the immune system (5-7).

Extracellular vesicles (EVs), which serve as a means of intercellular communication, between microorganisms and host cells, are produced by microbial communities that live inside vertebrate organs. They contribute to physiology and normal cell growth, transferring genetic information, which involves antigen presentation, immune suppression, and signal transduction, as well as the pathophysiology of various diseases, such as metastasis, tumor growth, and angiogenesis. In addition, they remove wastage from inside the cell. Inflammatory response-related EV functions are particularly crucial. Inflammatory illnesses like pneumonia, sepsis, and atopic dermatitis are caused by EVs produced from *Pseudomonas aeruginosa*, *Escherichia coli*, or *Staphylococcus aureus*, according to prior research.

Additionally, earlier studies have demonstrated that exosomes containing CagA from individuals with *H. Pylori* infection cause morphological abnormalities in AGS (adenocarcinoma gastric syndrome) cells and EVs produced from *H. Pylori* cause apoptosis. The sequential progression of gastric illnesses leading to gastric cancer has not yet been studied using metagenomic sequencing. Although *H. pylori* is known to generate EVs, nothing is known about how these EVs contribute to the etiology of gastric cancer. Extracellular vesicles (EVs) produced from *H. pylori-*infected gastric epithelial cells were found in blood samples from humans or animals and contained the *H. pylori* virulence factor A gene (*cagA*), indicating that they can deliver CagA into the circulation.

These results led some researchers to propose that *H. pylori* is implicated in the etiology of AS via EVs-based processes. Therefore, the therapeutic uses of EV in preventing *Helicobacter* disease have been discussed in this paper. *vacA* and related genes in different *Helicobacter* species originate from a common chromosomal gene with the *vacA* gene, which is present in all *H. pylori* strains. The complete *H. pylori* *vacA* gene encodes a 140 protein (Kilodalton to Atomic mass unit). The genus *Helicobacter* contains at least 20 distinct species, but only *Helicobacter cetorum* and *H. pylori*, a species isolated from the feces of marine mammals or the stomach, have intact *vacA* genes (6). In a manner similar to how gastritis and *H. pylori* (stomach inflammation) in humans are linked, gastritis and *H. cetorum* in cetaceans and possibly pinnipeds (seals) are linked (7, 9).

Genomic sequence analysis of *H. cetorum* strains from whales and dolphins revealed the presence of an intact *vacA* gene located near cysS (6), consistent with the association of *cysS* and *vacA* in *H. pylori*. The *H. pylori* *vacA* gene product shares 60–70% protein sequence identity with the proteins encoded by these *vacA* genes from *H. cetorum*, and another gene shares 66% identity. Additionally, the dolphin-isolated strain of *H. cetorum* contains a redundant triplet of divergent genes in *vacA* (6). It remains unknown whether the VacA proteins from *H. cetorum* have the same cytotoxic effects as those from *H. pylori*.

In *Helicobacter acinonychis*, a *Helicobacter* species isolated from cheetahs and other big animals, fragmented *vacA* pseudogenes were discovered (10, 11). Two almost identical fake *vacA* genes were discovered when the entire genome of one strain was sequenced (11). Following disruption of the *vacA* gene, familiar genes are likely to be translated. When recreated in-silico, the protein encoded by the *H. acinonychis* *vacA* pseudogene has around 64% similarity in amino acids with the closest match in *H. pylori* (12). *Helicobacter bilis*, *H. pylori*, *H. heilmannii*, *H. ailurogastricus, H. felis*, *H. bizzozeronii, H. suis, H. acinonychis*, and *H. cetorum* all contain "pseudo- are "*vacA*." Due to the resemblance among VacA and the "pseudo-VacA" gene products, this design is misnamed and is primarily restricted to the C-terminal end. Although it is not a component of the soluble VacA toxin, the C-terminal region is necessary for *H. pylori* VacA secretion (4, 8, 13).

*vlpC*, *faaA*, and *imaA* are the only *vacA*-like genes that have been empirically studied, as far as we are knowledge. These genes in *H. pylori* produce the proteins VlpC (VacA-like protein C, HP0922), FaaA (flagellar linked to autotransporter A, HP0609/0610), and ImaA (immune modulator of autotransporter A, HP0289) (10, 14). The transcription of each gene in the gastric media is compared to the transcription level during bacterial growth in vitro, and each of the *vacA*-like genes increases the ability of *H. pylori* to colonize the stomach in mouse models (10, 15).

VIC and pImaA are localized to the bacterial pole, while FaaA is located in the flagellum. *Helicobacter *VacA-like proteins are located on bacterial surfaces (10, 14, 16). Although knowledge about how these three proteins work is scarce, studying the mutant strain has revealed some information. In specific, analysis of the *faaA* mutant revealed inappropriate flagellum localization and decreased bacterial motility (14); interleukin-8 and TNF levels were higher in gastric epithelial cells co-cultured with the *imaA* mutant than in cells co-cultured with wild-type *H. pylori* (10); and mutations in *vlpC* are linked to increased metronidazole resistance (17).

## Transcription, Regulation and Secretion of VacA

About 120 nucleotides upstream of the AUG start codon, H. pylori vacA transcription begins (4, 17). Under stressful conditions, the structure of the stem-loop in the 5' untranslated region (UTR) of the vacA transcript (around nucleotide 51) stabilizes the vacA mRNA (18). The highest levels of transcription occur during the late log phase, and vacA transcription is regulated by the bacterial growth phase (19, 20). Although few studies have examined how environmental factors affect vacA regulation, some have shown that low pH, salt concentration, iron concentration, and bacterial interactions with host cells impact vacA transcription (18, 20, 21).

The 140 kDa protein produced from VacA translation is cleaved via Sec-dependent proteolysis, with additional carboxy-terminal proteolytic cleavage and removal of an amino-terminal signal sequence (8, 22). The cleavage yields an 88 kDa toxin, a peptide of about 12 kDa, and a protein of approximately 33 kDa (the β-barrel autotransporter) (3, 8).

The protease responsible for cleaving the carboxy terminus during proteolysis has not yet been identified. The 88 kDa toxin molecule traverses the outer membrane and can either remain on the bacterial cell surface or be released into the extracellular milieu as a soluble protein (together with the 12 kDa peptide) (2, 3). Secretion of the 88 kDa toxin is necessary for the 33 kDa autotransporter β-barrel to be incorporated into the outer membrane (8, 13). These characteristics suggest that an autotransporter or V-type secretion system is responsible for VacA release (4, 13, 22).

##  Formation of Membrane Channel by VacA

The majority of bacterial toxins that penetrate the host cells alter the cells by affecting intracellular enzyme activity, whereas the remainder alter the cells by creating pores in the plasma membrane of the host. Although it is known that VacA reaches host cells, it is still unknown whether or not it possesses enzymatic function. VacA is categorized as a pore-forming toxin because it may preferentially produce anions in flat bilayer lipids (23, 25). Bicarbonate, chloride, and other small organic molecules can be conducted by VacA channels, which function similarly to host cells' ClC channels Mutant VacA proteins show no vacuolating harmful action in cell culture tests and no ability to generate membrane channels in artificial bilayers (25, 26). 

Additionally, VacA channel activity in vacuolation in cell culture and lipid bilayers are disrupted by chemical inhibitors of Cl channels (27, 28). It should be highlighted that other cellular targets may also be affected by the pharmacological inhibitors utilized in these investigations in addition to VacA channels. The majority of the evidence suggested that the effect of VacA on host cells is related to the activation of endogenous cellular ion channels.

## Structure of VacA

When trypsin is present, and there is little proteolysis, the 88-kDa released VacA protein can be broken down into 33- and 55-kDa fragments or during long-term storage (8, 29). These are regarded as two VacA domains (*p33* and *p55*). The functionally active version of VacA can be recreated using recombinant combinations of *p55* and *p33* proteins (30, 31). The *p55* domain mostly has a beta-helical shape, and it can exhibit traits similar to those of the transmitter domains of a number of proteins secreted by gram-negative bacterial species' autotransporter mechanism. For the *p33* domain, there are no high-resolution structural data available (32).

The 88 kDa VacA secretory protein's amino acid sequence is not strongly connected to any other known bacterial toxin's sequence. When VacA is introduced to the cell exogenously, both *p33* and p55 are necessary for the toxin to effectively bind to the plasma membrane (33). To construct selective anion channels, the *p33* domain must be inserted into membranes (25, 26). The minimal area needed by VacA for vacuolation when it is produced intracellularly spans 1-422 residues, including the whole *p33* domain plus 111 amino acids from the amino-terminal section of the *p55* domain (34, 35).

The expected hydrophobic region within the membrane-spanning VacA length is represented by a 32 amino acid uncharged sequence at the N terminus of the *p33* domain (28). A VacA mutant lacking cell vacuolation activity and deficient in the creation of membrane channels in planar lipid bilayers is produced by the deletion of this region (25). Three tandem membrane-spanning motifs, designated as glycine residues at locations 14, 18, 22, and 26, may be found in the amino-terminal hydrophobic region of VacA (26, 28, 36). The development of the VacA channel and vacuolation activity are stopped by mutations or mutations of amino acids in this area, such as glycine residues at positions 14 and 18 or proline residues at position 9 (26).

In solution, VacA oligomerizes to form structures resembling a flower or a snowflake (37, 38). These assemblies include monolayer structures—primarily hexamers and heptamers, although higher-order forms are occasionally present—as well as bilayer structures (tetradecamers and dodecamers). According to some data, VacA activity depends on oligomerization (39–41). The membrane channel structure of VacA is similar to that of the water-soluble, monolayer VacA oligomer. VacA oligomers dissociate into monomers when exposed to either acidic or alkaline pH (40).

**Fig. 1:**
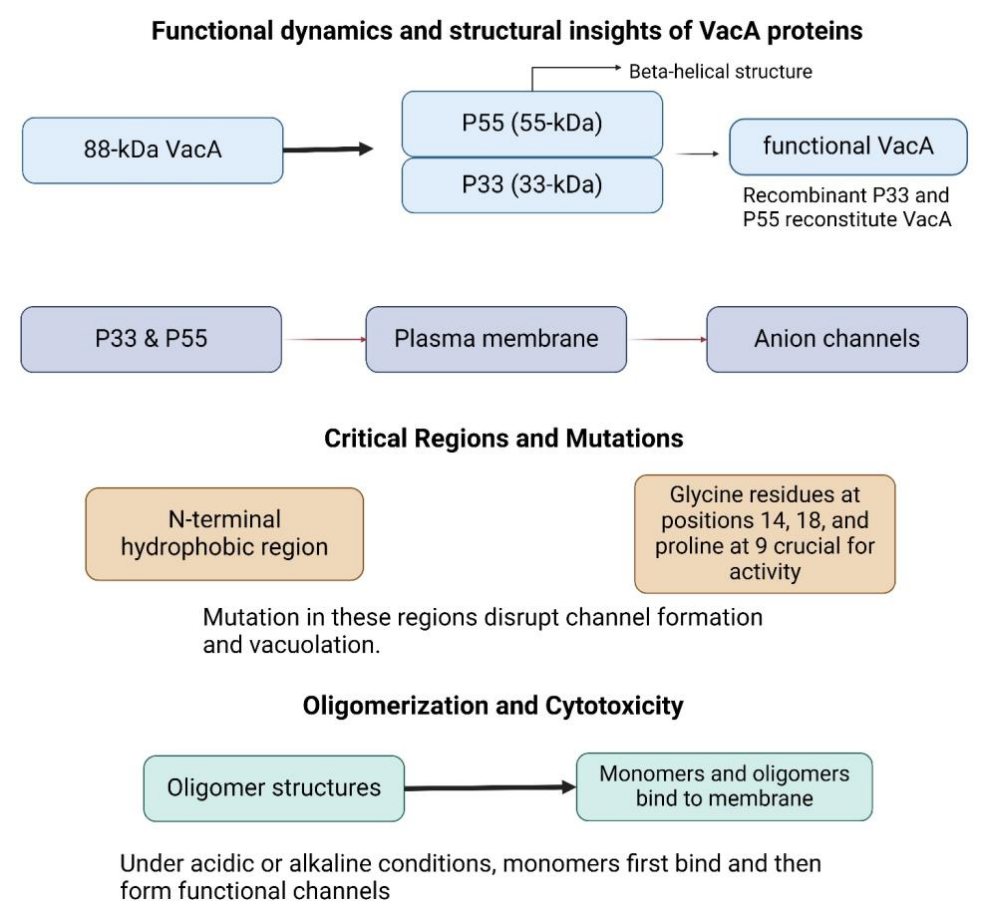
Functional dynamics: VacA requires precise structural domains and oligomerization for its cytotoxic function, forming channels in the host membrane under specific conditions. The VacA protein can be cleaved into P55 and P33 fragments, essential for its activity. Both P33 and P55 are required for binding to the plasma membrane and anion channel formation.

Compared to preparations of VacA oligomers, those exposed to low or high pH significantly increase cytotoxic activity when added to cultured cells (42, 43). Hence, it is hypothesized that VacA initially binds to the host cell's plasma membrane as a monomer and, upon oligomerization, transforms into a functional membrane channel (Figure 1).

## VacA Diversity Among H. pylori Strains

The toxin vacuolation activity of different *H. pylori* strains vary significantly, according to preliminary studies (6). The variation in VacA transcription between strains is one of the significant factors linked to this problem (44). The amount of VacA protein secreted varies between strains as well (3). As of right now, it is still unclear whether the variation in VacA protein secretion between strains is primarily caused by variations in VacA transcription, differences in the stability of the transcripts, or variations in the efficacy of the various forms of VacA that are secreted. All *H. pylori* strains have an *H. pylori* gene, and the *vacA* open reading frame is present in the majority of strains. A small percentage of isolates have *vacA* frameshift mutations, which prevent the production of VacA protein (45).

Numerous diverse groups of *vacA* alleles exist, each with a unique regional or geographic distribution, according to phylogenetic analyses of the *vacA* sequences from several of *H. pylori* strains (12). For instance, the majority of *H. pylori* strains recovered from East Asia have *vacA* alleles that differ markedly from those present in most *H. pylori* strains obtained from Europe or Africa. are unique (12, 46). The favorable sequence alterations in the *vacA* region that codes for the *p55* domain and are connected to the surface exposure location in the *p55* structure of crystal are mostly to blame for the variation among the *vacA* alleles (12).

Signal sequence region (s-region), middle region (m-region), and intermediate region (i-region) are the three primary regions of variation in the VacA sequence that are known (43, 47). Sequences can be divided into two primary categories between each of these regions: s1 or 2s, m1 or m2, and i1 or i2). The secretory toxin's amino terminus and an amino terminal signal sequence are both located in the s-region of VacA (43, 48, 49). The VacA’s p33 domain contains the i-region (47). A portion of the second *p5* corresponds to the m region. Due to the frequent occurrence of homologous recombination in *H. pylori*, *vacA* alleles may contain many conceivable combinations of m, s, and I region types (s2-i2-m2, s1-i1-m1, etc.) (43). The s2 form of VacA cannot induce vacuolation in mammalian cells, in contrast to the s1 form (48, 49).

Different positions of signal sequence cuts in the s2 and s1 proteins of VacA are the cause of this variance in activity. Particularly, the s2-type protein contains an N-terminal 12 amino acid hydrophilic region while the s1-type protein does not (43, 48, 49). According to several research, VacA's m1 and m2 types exhibit different cell-type characteristics. For instance, m1 VacA activity has a stronger affinity for HeLa cells than m2 VacA does, although both m2 and m1 forms are substantially activated by RK-13 cells (50, 51). VacA binding to cells varies depending on the cell type, and channel creation properties may also play a role in the difference in VacA m2 and m1 proteins' activities (50, 51). VacA's m region has a 148-residue fragment that has been identified as being cell type specific (51).

According to a study, in strains that produce s1-m2 forms of VacA, the I region (located within the p33 domain) determines the toxin vacuolating activity (8). In tests using Jurkat T cells, the i1/VacA forms were found to be more active than the i2/VacA forms (13). The risk of *H. pylori* gastric diseases was linked by epidemiological studies to some of the vacA alleles found in *H. pylori* strains. Infected people with strains containing VacA type s1, i1, or m1 have a higher risk of developing stomach cancer or peptic ulcer disease than infected people with strains containing VacA type s2, i2, or m2 (43, 47, 53, 54).

The impacts of VacA as well as the impacts of other virulence factors are both linked to the elevated disease risk seen with strains bearing the m1, i1, or s1 variants of VacA. In example, strains with the s1 allele of *vacA* typically include the outer membrane adhesion protein BabA and the cag pathogenicity island, which encodes several crucial virulence factors, including the type four secretion system and CagA. They frequently lack *babA* and typically lack the *cag* pathogenicity island (54).

## Effect on Epithelial Cells

### Endosomal Changes

The majority of research focuses on VacA's capacity to cause vacuolation in cultivated cells. Much research on this phenomenon and other VacA activities have used the protein's highly active version, m1-i1-s1. After adding VacA to the cells, vacuolation can be seen after a few hours and is enhanced even with weak bases (55). Vacuoles are produced from late endosomal components because the membranes of VacA-induced vacuoles contain indicators typically present in the membranes of late endosomes (LEs) (56, 57). The secretory monomeric form of VacA, according to functional models of VacA-induced vacuolation, binds to the plasma membrane. In the LE membrane, anion-selective channels are created by the exchange of VacA monomers with Les after binding (25, 29

**Fig. 2:**
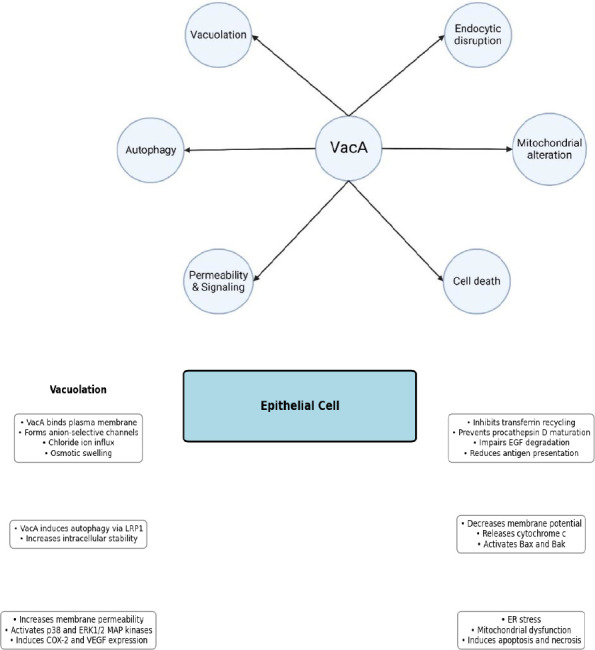
Effect of vac toxin on epithelial cell.

Chloride ions are transported through VacA channels in LE membranes, increasing the concentration of chloride inside the channel. This rise in chloride concentration then increases the V-ATPase proton pump’s activity and lowers the pH inside the channel. Les, which are protonated and confined in an acidic environment, are formed when weak membranes diffuse into them. Les has osmotic swelling as a consequence, which causes cellular vacuolation (58, 59). Further to generating cell vacuolation, VacA also disrupts the normal endocytic component exchange, which results in some functional alterations. It also prevents transferrin recycling, the development of procathepsin D, the intracellular degradation of epidermal growth factor, and the presentation of antigens in immune cells (Figure 2) (60-62).

## Autophagy

The controlled breakdown and cellular components’ recycling in the cytoplasm is known as autophag, and it occurs when VacA is introduced to cultivated gastric epithelial cells. In *H. pylori*, VacA is both required and sufficient for autophagy. The VacA capacity to create membrane channels is also required for VacA-induced autophagy, which is similar to VacA-induced vacuole formation. However, autophagosome production in response to VacA is more frequent and larger than intracellular vacuoles that respond to VacA (63). The binding of VacA to the low-density lipoprotein receptor-related protein 1 (LRP1) is required for VacA-induced autophagy, even though the processes by which VacA causes autophagy are not entirely understood (64).

Increased intracellular VacA stability and cellular vacuolation are caused by autophagy inhibition. The initial reaction of the host cells to the breakdown of VacA and to avoid cell damage from toxin formation is thought to be caused by the autophagy induced by VacA (63). Although short-term acute exposure to VacA stimulates autophagy in host cells, this exposure affects autophagy over time (65, 66).

When cells are treated with VacA, a number of mitochondrial alterations occur, including a decrease in the potential of the mitochondrial membrane, the release of cytochrome c, the activation of Bax and Bak, and the fragmentation of the mitochondria (67-69). It has been suggested that VacA directly affects mitochondria since it can localize in mitochondria after entering host cells. The fact that VacA can lower the membrane-isolated mitochondria’s potential and that it can permeate the inner mitochondrial membrane (IMM)is evidence in favor of this concept. VacA channel activity is required for VacA to cause mitochondrial dysfunction (68, 69).

As a result, their idea was that VacA reaches the mitochondria, where it lowers the transmembrane potential and potentially even forms pores. The initial release of cytochrome c, Bak and activation of Bax, and further Bax/Bak-dependent cytochrome c release are all stimulated by this depolarization. Another possibility is that VacA directly causes the mitochondrial malfunction it is known to induce. To promote mitochondrial apoptosis, for instance, VacA may indirectly function by activating proapoptotic factors (67).

VacA causes epithelial cell vacuolation, disrupts endocytosis, induces autophagy, and affects mitochondria. It increases cell permeability and activates signaling pathways, leading to COX-2 and VEGF expression. VacA can also induce cell death, highlighting its role in *H. pylori* pathogenesis.

**Fig. 3:**
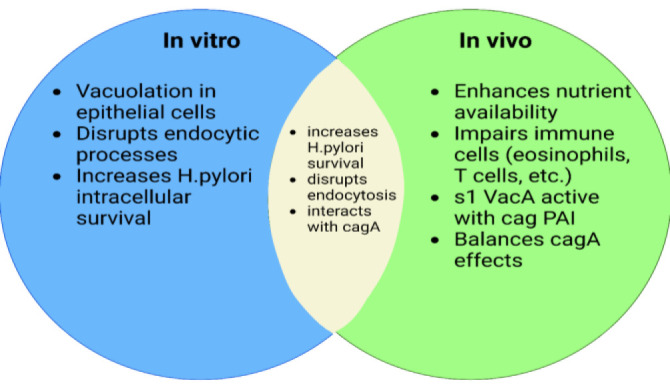
Comparison of VacA Activities in Laboratory and Living Conditions

## Epithelial Barrier Changes

VacA enhances the permeability of the plasma membrane in cultivated epithelial cells, causing different small molecules and anions, such as urea, bicarbonate and chloride to flow into the extracellular space (27, 70). The creation of VacA channels in the plasma membrane is thought to be the cause of the permeabilization of cells caused by VacA (23, 39, 70). VacA not only makes the plasma membrane more permeable, but it also makes the polar monolayers of neighboring cells more permeable. We still don't fully understand the process by which VacA increases the permeability of nearby cells (70, 71).

## Change in Cell Signaling

After cells are exposed to VacA, several cellular changes can be promptly observed. These changes are likely the result of VacA adhering to the host cells' surfaces. A MAP kinase called VacA stimulates p38 in both T cells and gastric epithelial cells (72, 73). Cyclooxygenase 2 (COX-2) expression is induced as a result of VacA activating the *p38* signaling pathway, which also results in an increase in prostaglandin E2 synthesis. Transcription factor 2 (ATF-2) can also be activated by VacA-induced stimulation of the *p38* signaling pathway (73).

VacA can also make ERK1/2, a different MAP kinase, active a signaling pathway that activates the G protein-interacting receptor-coupled kinase (Git1) and results in the overexpression of vascular epithelial growth factor (VEGF) can also be activated by VacA in addition to MAP kinases. And beta-catenin takes over the signaling pathway (74). Although the VacA receptor RPTP-beta has been reported to be necessary for Git1 activation, and the epidermal growth factor receptor has been reported to upregulate VEGF, the receptors of cell surface of VacA required for the activation of the majority of these pathways have not yet been identified. It is essential (74, 75).

## Cell Death

Although cellular vacuolation brought on by VacA does not result in cytolethality, exposure of epithelial cells to VacA has the potential to cause cell death (77-79). The duodenal AZ521 cells are particularly vulnerable to VacA-induced cell death (74, 70). A number of mitochondrial modifications occur prior to VacA-induced cell death, indicating that these changes are mechanistically significant in the process by which VacA induces cell death. VacA is responsible for cell death because it decreases the production of pro-survival factors and stresses the endoplasmic reticulum (ER). Additionally, VacA can kill cells by necrosis and apoptosis (74).

## Effect on Immune Cells and Parietal Cells

### Effect on Immune Cells

Numerous immune cell types, including dendritic cells, mast cells lymphocytes, eosinophils, and macrophages, can have their functions altered by VacA (81-83). VacA can prevent B cells from presenting an antigen by inhibiting B cell and T cell activation and proliferation. Megasome development is encouraged in macrophages by VacA, which also interferes with the maturation and operation of other vesicular components (82). In addition to altering many signal transduction pathways in macrophages, VacA can also lead to apoptosis in these cells (83). These VacA-induced side effects may reduce macrophages' capacity to absorb *H. pylori*. VacA suppresses the immune system and increases the expression of the pro-inflammatory enzyme R in neutrophils and macrophages (37).

### Effect on Parietal Cells

According to two studies, VacA prevents parietal cells from secreting stomach acid. In one investigation, parietal cells exposed to VacA experienced plasma membrane permeabilization and calcium influx, which ultimately disrupted the organization of actin in apical microvilli and inhibited acid secretion. At the moment, it is unclear if the of VacA on parietal cells is related to the decrease in stomach acid output which is occasionally noticed when *H. pylori* infection is present (84).

### Connection of Cell Surface and Receptors

Regarding the saturation or lack of saturation of VacA binding to cells, many investigations have reported varying results. Thus, it is unknown whether VacA binds to abundant, a single receptor, a receptor with low affinity, or a number of cell surface components. There have been reports of several fictitious VacA receptors on the surface of epithelial cells, including both lipid and protein receptors. These contain phospholipids, sphingomyelin, glycosphingolipid, heparan sulfate, receptor protein tyrosine phosphatase alpha and beta (RPTP), epidermal growth factor receptor (EGFR), low-density lipoprotein receptor-related protein 1 (LRP1) (64, 85).

Sphingomyelin is the only part of these putative receptors whose presence or absence is a crucial functional factor in determining how sensitive epithelial cells are to VacA as well as whether VacA binds to the cell surface (86). The toxin's location in lipid rafts is likely caused by the binding of VacA to sphingomyelin (87). The integrin 2 subunit (CD18) is the only VacA receptor found on T cells, despite the fact that several putative VacA receptors have been discovered on epithelial cells (88). Damage to gastric tissue results from altered cell signaling brought on by VacA bound to RPTP beta. Because of this, giving VacA to wild-type mice orally causes gastric damage, whereas giving VacA to RPTP-deficient or RPTP-beta knockout mice does not cause gastric damage. An RPTP-null or RPTP-beta knockout mouse model has shown that VacA is internalized within epithelial cells and that RPTP-beta is not the only receptor for VacA (75). VacA-induced autophagy and apoptosis depend on VacA bound to LRP1 (64).

### Pore Formation on the Cell Surface

Following cell surface binding, VacA enhances plasma membrane permeability and induces membrane depolarization (36,39). The creation of a selective membrane anion channel and the insertion of VacA into the plasma membrane are thought to be responsible for these modifications. suggested that this toxin can create channels inside of cells (mitochondria and endosome). The connection between intracellular toxin exchange and VacA channel development is poorly understood (32–36).

### Internalization of VacA and Intracellular Exchange

Cdc42-dependent, a clathrin-independent, and Rac1-dependent pathway internalizes VacA after it binds to the cell surface, and this process is necessary for actin polarization (89, 90). Within 10 minutes of VacA internalization, GEECs enriched in glycosylphosphatidylinositol-anchored protein (GPI-AP) were identified, followed by EEs in 30 minutes and Les in two hours. Numerous investigations have shown that VacA is not only found in endosomal components within the cell. For instance, VacA has been discovered in the host cell in connection with mitochondria. The exact mechanism by which VacA translocates to mitochondria is unknown. According to the scenario they put up, VacA is delivered straight from the endosome to the mitochondria by the VacA subunit that contains the endosome (87).

This hypothesis is supported by the finding that VacA’s effects on cells occur through the simultaneous destruction of endosomes and mitochondria. Another proposed scenario is that VacA is released into the cytosol and then transported into the mitochondria via a protein-mediated mechanism (71, 72). It remains unknown whether VacA, when supplied exogenously, can eventually access the cytosol—although the cell benefits when VacA is expressed in or injected into host cells (either directly from the plasma membrane or by diffusion from endosomes). Some studies have demonstrated that VacA may migrate retrogradely via the ER and Golgi, although further research on this matter is still pending. Additional studies are needed to determine the intracellular trafficking of VacA (34 91).

### VacA Activity in Animal Models

The potential contribution of VacA to the expansion of *H. pylori* in mammal stomachs is being studied using animal models. Mice, gnotobiotic piglets, and gerbils can clone the VacA mutant strain of *H. pylori*, proving that VacA is not required for gastric cloning. The VacA mutant strain, however, has a competitive disadvantage when VacA mutant and isogenic wild-type strains are co-infected. Additionally, in comparison to the wild-type strain, the *vacA* mutant strain clones less well or at a lower rate (92, 93).

Animal model experiments have revealed a role for VacA in advancing gastric disease. Purified VacA administered orally or intragastrical to mice damages the activated inflammatory cells and the gastric mucosa (75, 94) It has been established that VacA is connected to the etiology of gastric ulcers in humans based on the ability of VacA to cause ulcers in mice when injected into the stomach. The VacA’s intragastric concentration in animals that received intragastric VacA was much greater than the concentration of 20-800 based on the vertebral ELISA approach used on a sample of patients with *H. pylori*. The estimated pg per Ml VacA is comparable to that found in gastric juice, but the local VacA concentration at the areas where *H. pylori* interacts with gastric epithelial cells may be substantially higher than that seen in gastric juice (93).

The vacA mutant strain produced fewer stomach ulcers than the isogenic wild-type strain in experiments on rodents infected with *H. pylori* (95). Additionally, cloning studies in mice revealed that *H. pylori* strains producing an active form of VacA (s1-i1) were more effective than strains producing an inactive form of VacA. (s1-i1). The stomach's metaplasia and inflammation are more severe and pervasive (s2-i2 or s1-i2) (93). According to a different study, the DvacA mutant caused more severe gastric pathology in mice and induced stronger T-helper 17 (Th17) and T-helper 1 (Th1) responses when compared to the wild-type strain. The immunomodulatory properties of VacA may explain the latter finding (96).

*H. pylori* infection in humans frequently results in the production of gastric and serum mucosal antibody responses to VacA, but this hormonal immune response clears the infection. It doesn't for *H. pylori*. Animals immunized with VacA, on the other hand, acquire protective immunity against future *H. pylori* challenges (97-99). Vaccines used for therapeutic immunization, i.e., immunization intended to speed up the clearance of *H. pylori* infection, also contain VacA as a component (100).

Asthma and allergies are inversely correlated with *H. pylori* infection in people (101). The protective effect of *H. pylori* is ascribed to the tolerogenic reprogramming of dendritic cells, according to studies done in a mouse model showing that the infection protects against the emergence of allergic asthma (102, 103). VacA is necessary for protection against allergic asthma in animal models, and it has been linked to the ability of *H. pylori* to elicit tolerogenic impacts on mouse dendritic cells in vivo and in vitro (104).

### Comparison of VacA Activities in Laboratory Conditions and in Living conditions in the body

In vitro, VacA causes a variety of changes in various cell types. It is conceivable that some of VacA's in-vitro activities are more prevalent in vivo. As a result, VacA activities are more closely linked to an increase in gastric *H. pylori* colonization or the emergence of conditions such as gastric cancer or ulcers. One of the most extensively investigated occurrences under laboratory circumstances is cell vacuolation caused by VacA.

Gastric epithelial cells of humans are susceptible to the effects of VacA , and vacuolation has been reported occasionally in gastric biopsy samples of epithelial cells, albeit this phenomenon is less pronounced in cultured cells that have been exposed to VacA. According to a study, VacA can prolong *H. pylori*'s intracellular survival in gastric epithelial cells(105, 106). *H. pylori*, in contrast, does not multiply inside intracellular vacuoles and is largely an external organism (106). Therefore, it is challenging to foresee the process by which gastric *H. pylori* colonization or *H. pylori*-related gastric illnesses are caused by vacuole formation. On the other hand, the bacteria may benefit from alterations in the host cells brought on by VacA's interruption of endocytic exchange (for instance, by limiting the presentation of antigens) (62).

Enhanced availability of vital nutrients or growth stimulants (such metals), which results in increased proliferation of *H. pylori*, is a possible action for VacA in vivo. Both VacA-induced alterations in paracellular permeability and VacA channels in the plasma membrane can release nutrients. According to research, recirculating VacA disturbs transferring in host cells, increasing iron availability and promoting *H. pylori* growth on epithelial cell surfaces. VacA-induced cell death might cause the release of nutrients.

VacA can significantly influence the activity of toxins inside the body by impairing the function of immune cells in laboratory settings. By interfering with the normal operations of eosinophils, T cells, neutrophils, B cells, and macrophages, in particular, VacA can lessen the host's immune response and promote the survival of *H. pylori* stomach colonization.

The s1 version of VacA, which is more active in vivo and frequently contains the cag pathogenicity island, is produced by *H. pylori* strains, as opposed to the s2 form, which frequently does not. A type 4 secretion system is necessary for CagA entry into host cells, and cag PAI encodes a CagA effector protein that induces a number of alterations in host cells. A functional relationship between cag PAI and VacA products is suggested by the co-selection of the s1 *vacA* and cag PAI variants and by the similarity in the phylogenic structure of the *cagA* and *vacA* genes (12). Several investigations supported this idea by demonstrating that CagA and VacA both partially block each other's actions and exchange when they are present in vitro. As a result, another significant function of VacA in the body is that it may act as a beneficial counterbalance to CagA (Figure 3) (107-109).

### H. pylori EVs and Pathogenesis

EVs, which are nano-sized membrane-bound structures, play many roles in *H. pylori* pathogenesis. In the first step, EVs are involved in the attachment of bacteria to host cells and tissues. This target is achieved by increasing the attachment of bacteria to host cells, and for this reason, EVs, along with other colonization factors such as adhesives and flagella, play an important role in intestinal colonization (106). *H. pylori* EVs contain adhesive elements such as BabA and SabA. Also, the presence of urease in EVs helps to regulate the pH of the stomach and the establishment of bacteria (111).

In the next step, the continuous release of EVs, which contain various biomolecules such as proteins, lipids, nucleic acids (especially micro-RNAs and non-coding RNAs), and virulence factors, induces and perpetuates inflammation in the stomach environment. By increasing the absorption of neutrophils and the destruction caused by inflammation, more suitable sites are provided for the establishment of more bacteria. Therefore, EVs are also effective in the most important stage of persistent infection (112). 

On the other hand, EVs are also a way to release two important pathogenic factors of *H. pylori*, CagA and VacA. Most CagA-carrying EVs affect cell junctions and host regulatory proteins and have oncogenic functions, while VacA-carrying EVs exert their effect by inducing the production of inflammatory cytokines (113). Therefore, these virulence factors not only protect against destruction by the host's immune system and are delivered to host cells during transport coverage but can also spread to other parts of the body and form systemic destructive effects. This is why EVs of *H. pylori *are also related to other diseases, such as atherosclerosis (114). Moreover, EVs containing CagA have been isolated from the serum of patients colonized with *H. pylori* (115). Also, the association of *H. pylori* with liver diseases has been confirmed as a risk factor for the occurrence and progression to cirrhosis and liver carcinoma (116). Also, the increased risk of pancreatic and colorectal cancers, Parkinson's disease, Alzheimer's disease, chronic obstructive pulmonary disease (COPD), diabetes mellitus, inflammatory bowel disease (IBD), and idiopathic thrombocytopenic purpura in patients' colonized with *H. pylori* is also related to the release of virulence factors by EVs (117, 118).

### H. pylori EVs and Host-Cell Interactions

EVs' release and function depend on the interaction with the host cell. On the other hand, *H. pylori* EVs affect the performance of the host. This effect may be through direct contact with the cell surface or fusion with the plasma membrane. The mechanism depends on the target cell that determines the type of interaction. Therefore, *H. pylori* EVs increase the proliferation of epithelial cells by influencing host cell signaling pathways and potentially causing gastric cancer. High levels of EVs *H. pylori* in the gastric juice of patients with gastric cancer have been confirmed in studies (119, 120). Other consequences of the induction of signaling by *H. pylori* EVs are the gastric epithelium's deformation and the cells' stretching. There is still no consensus whether the CagA oncoprotein is responsible for this change or whether it is a direct result of its interaction with the cell (121). On the other hand, some studies have shown that *H. pylori* EVs induce apoptosis in gastric epithelium. This action seems to be done by inducing increased expression of HSP 60 in the host epithelium. Also, the protein plays an important role in suppressing the immune response to the tumor (122, 123). Another mechanism is the increasing of the expression of FRA-1, a transcription factor complex subunit activator protein-1, by *H. pylori* EVs. FRA-1 plays an important role in increasing cell proliferation and migration in the stomach tissues (124). Furthermore, VacA-carrying EVs *H. pylori* induce apoptosis in the host cell by affecting cytochrome oxidase and engaging the Fas-FasL pathway. In another way, EVs carrying VacA can induce the accumulation of connexin-43 in cells and induce the process of autophagy and, ultimately, cell apoptosis by the ERK/Ras1 pathway (113, 125).

EVs production from infected host cells often occurs due to changes in the calcium gradient or effects on GTPases, especially Rab27. These EVs contain binding proteins such as integrins that facilitate their interaction with other host cells. In addition, they can carry different types of *H. pylori* pathogenesis factors. The effect of host EVs on reducing immune responses, inducing proliferation of cancer cells, and promoting angiogenesis increases the complications caused by *H. pylori* infection (114). Some studies have also confirmed that EVs originated from gastric cells of people colonized with *H. pylori* containing CagA and other virulence factors of this bacterium. The EVs are absorbed by liver cells and increase the proliferation and migration of these cells by activating NF-κB and PI3K/AKT signaling pathways. Perhaps this is one of the important reasons for the connection between *H. pylori* and liver cancer (126).

### H. pylori EVs and Host Immune Response

Modulating the host's immune response and interacting with the microbiota are other ways that *H. pylori* EVs interact with its host. At first, biomolecules transported and released by EVs are all except microbial pathogen-associated molecular patterns (PAMPs). Therefore, they are involved in the activation of the immune response and the development of infection. Also, these biomolecules play a key role in the interaction of bacteria with host cells. *H. pylori* EVs induce the production of many inflammatory cytokines such as TNF-α, IL-1β, and IL-6 (127). A probable cause of this phenomenon is the release of phosphorylated mesenchymal-epithelial transfer factor (MET) from gastric cancer cells under the influence of *H. pylori* EVs. MET is then absorbed by macrophages and increases the expression of inflammatory cytokines in them (128).

Therefore, host cells are infected with *H. pylori,* releasing EVs that play a role in the pathogenesis of this bacterial infection. Another example is EVs isolated from bacterial infected macrophages, which contain micro-RNAs and induce the expression of components involved in inflammatory responses such as IL-23, MHC-I, and CD-40 (129). Also, EVs induce the expression of MHC-II on the surface of gastric epithelial cells, which is a way to induce apoptosis in them and the progression of *H. pylori* disease (130). Apoptosis can reduce the deleterious effects of the inflammatory response, and when it occurs in lymphocytes or antigen-presenting cells, it can reduce the effectiveness of adaptive immune responses against this bacterium. Hence, *H. pylori* EVs can drive host immune function towards chronic responses (131). In addition, in animal studies, the production of interferon-γ, immunoglobulin-G, and IL-17 was also increased due to EVs. But T cell activity is suppressed by EVs (120). One of the causes of changes in gene expression and signaling in the host cell by *H. pylori* EVs is the presence of non-coding RNAs.

Studies showed EVs *H. pylori* micro-RNAs are one of the reasons for changes in T-lymphocyte function because of their ability to present by antigen-presenting cells. Also, micro-RNAs modulate the host's inflammatory response and the survival of bacteria in the gastric mucosa (132). An example is miR-155, which reduces the killing activity of macrophages against *H. pylori* by reducing MyD88 and NF-kB signaling (133). Another example of micro-RNA interactions with the host immune system is miR-25, which affects cell proliferation, apoptosis, and cytokines by inducing TNF-α production. Therefore, miR-25 seems to play an important role in the occurrence of cell proliferation in *H. pylori*-related cancers (134). Also, *H. pylori* EVs induce IL-10 production from monocytes and suppress the immune response (135). 

*H. pylori* colonization increases the pH of the stomach and affects the epithelium of the tissue, facilitating the establishment of other microorganisms. For this reason, changes in the microbiota of the stomach and parts of the intestine have been confirmed, especially in *H. pylori*-related cancers. This dysbiosis itself has a positive effect on the progress and severity of the disease (136). In healthy people, the largest population of stomach microbiota includes *Actinobacteria*, *Bacteroidetes*, *Firmicutes* and *Fusobacteria*, respectively. But with the predominance of *H. pylori* population in the stomach, some types of non-*H.* *pylori* also become stomach microbiota (137). Transport and release of urease, induction of inflammation, recruitment of neutrophils and subsequent modulation of the immune response, release of virulence factors especially CagA and VacA by *H. pylori*-derived EVs discussed earlier all contribute to dysbiosis in gastrointestinal tracts (138). Despite the emphasis of many articles on the importance of EVs *H. pylori* in the pathogenesis and regulation of immune system function, there are still many challenges to target these structures for treatment and insufficient information is provided in this field.

### Concluding Remarks

As a result, when these bacteria are infected, *H. pylori* continuously sheds outer membrane vesicles from their surface. Because they serve as carriers for the transmission of virulence factors, *H. pylori* OMVs may be crucial in the pathogenesis of the infection due to their makeup and capacity to pass the gastric barrier into the intestines and circulation. Locally, OMVs can direct effects like cytotoxicity, apoptosis, or tight junction disruption, or they can indirectly cause immune cells to release cytokines, proteases, or tissue-damaging chemicals like MMPs or ROS.

They may attract and present M cells to other immune cells as a result of their passage from the gastric niche via the digestive tract to the intestine, which could then result in immunomodulation of immunological mechanisms and the release of OMVs into the bloodstream. The significance and processes by which OMVs produced by *H. pylori* promote inflammation or tolerance may be explained by studies on their role in the etiology of extra-gastric illnesses such coronary heart disease or food allergies.

But what can be concluded in this article is that VacA, a secreted bacterial toxin, has been significantly identified and defined, which are different in terms of amino acid sequence and intracellular exchanges and actions f different cells are susceptible to VacA under laboratory conditions, and this toxin can cause extensive cellular changes, as mentioned above, VacA promotes the ability of H. pylor to increase cloning in the body, and finally, it contributes to the pathogenesis of diseases induced by *H. pylori.*
